# Job Satisfaction in the Long-Term Care Workforce: How Ageism Plays a Role

**DOI:** 10.1093/geroni/igaa057.1236

**Published:** 2020-12-16

**Authors:** Alex Hennessa, Tracey Gendron, Verena Cimarolli, Jennifer Inker, Annie Rhodes, Robyn Stone

**Affiliations:** 1 LeadingAge, Washington, District of Columbia, United States; 2 Virginia Commonwealth University, Richmond, Virginia, United States

## Abstract

Prior research has demonstrated that ageism, specifically negative attitudes and behaviors about growing old, can be barriers to delivering high-quality long-term care (LTC), but little is known about how ageism may be related to job satisfaction – an important driver of workforce retention in LTC. Hence, the purpose of this study was to examine the role of ageism in job satisfaction in LTC. Our cross-sectional study used data collected from 265 staff members of aging services organizations (e.g. nursing homes, assisted living) representing the continuum of job types in LTC. The study examined the relationship between ageist attitudes (i.e. internalized and relational aging anxiety; affinity for older persons) and ageist behaviors, and job satisfaction when controlling for socio-demographic (i.e. age; gender; ethnicity) and employment-related variables (i.e. years of employment; advanced training in gerontology; direct care vs. managerial position). Results of a regression analysis showed that lower internalized aging anxiety and higher affinity for older people were significantly associated with higher levels of job satisfaction. Findings suggest addressing ageism to improve job satisfaction in LTC and provide some evidence for incorporating ageism screening and training into recruitment and onboarding of staff to enhance job satisfaction and to mitigate turnover.

